# A new treatment for blown-out myotomy, a diverticulum formed after peroral endoscopic myotomy

**DOI:** 10.1055/a-2226-9356

**Published:** 2024-01-23

**Authors:** Hiroya Sakaguchi, Shinwa Tanaka, Hirofumi Abe, Douglas Motomura, Hitomi Hori, Takashi Toyonaga, Yuzo Kodama

**Affiliations:** 1 592910 Division of Gastroenterology, Department of Internal Medicine, Kobe University Graduate School of Medicine, Kobe, Japan; 2 Tanaka Internal Medicine Clinic, Kobe, Japan; 3 8166 Gastroenterology, The University of British Columbia, Vancouver, Canada

Blown-out myotomy (BOM) is a potential postoperative complication for achalasia patients after both surgical and peroral endoscopic myotomy (POEM). It causes diverticular-like changes and food retention in the lower esophagus due to residual contraction and impaired discharge of esophageal contents. It occurs in 17.8% of patients after POEM and can be symptomatic 1 ; however, no effective treatment for patients with symptomatic BOM has been reported.

A 48-year-old man who underwent POEM for esophageal achalasia (Chicago classification type II, straight type, dilation grade 1) developed BOM 5 years after the procedure. Esophagogastroduodenoscopy (EGD) showed retention of food residue and diverticular-like changes in the lower esophagus ( Fig. 1 **a** ). A newly developed septum was seen in the former myotomy line, just above the esophagogastric junction, and was likely contributing to his symptoms ( Fig. 1 **b** ). An endoscopic septal myotomy was therefore planned.

**Fig. 1:**
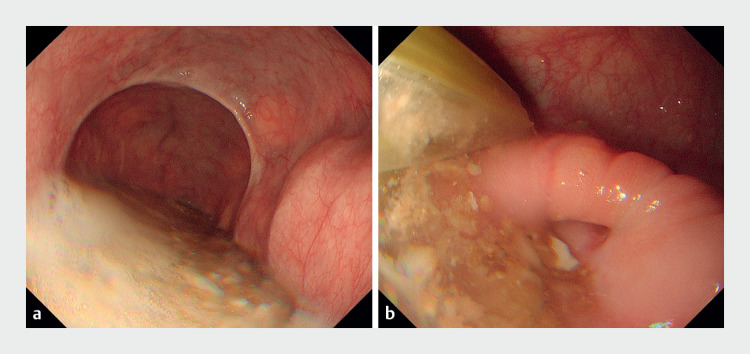
Endoscopic images of the diverticulum prior to treatment showing: **a** retained food residue; **b** the newly formed septum.

A mucosal incision was made in the lower esophagus in the 7-oʼclock position, avoiding the former myotomy line. A submucosal tunnel was created and continued obliquely through the former myotomy line into the stomach. The septum, which was composed of muscle tissue, was exposed during the submucosal tunneling, and septal myotomy was then performed ( Fig. 2 ). The wall of the diverticulum was flattened and the entry site was closed ( Fig. 3 ; Video 1 ).

**Fig. 2:**
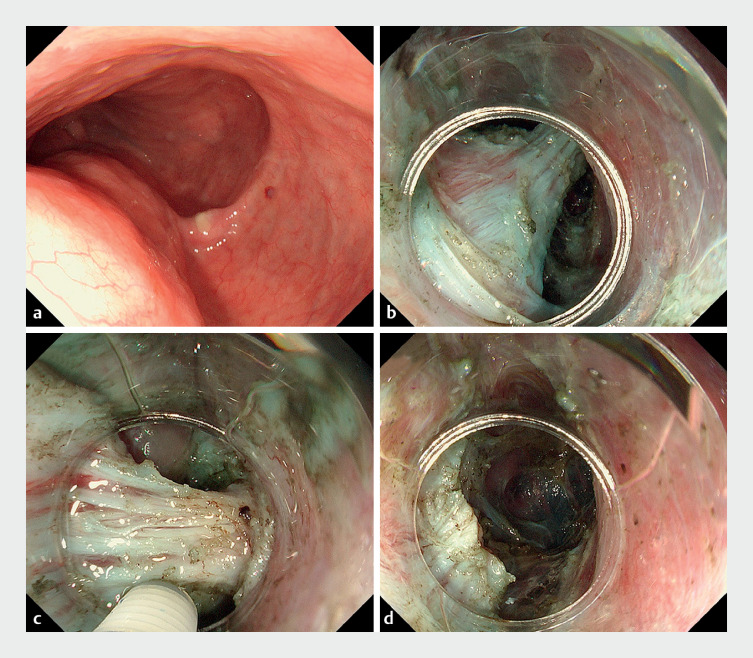
Endoscopic images of the blown-out myotomy being treated showing: **a** the diverticulum formed after peroral endoscopic myotomy; **b** the exposed muscular layer that serves as the septum on the anal side of the diverticulum; **c** the myotomy being performed; **d** the completed myotomy.

**Fig. 3:**
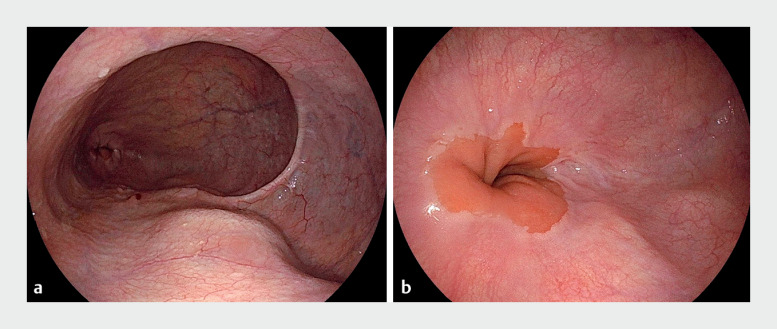
Endoscopic images of the appearance after treatment showing: **a** no food residue; **b** the flattened wall of the diverticulum.

A new treatment is performed for blown-out myotomy, a diverticulum that forms after peroral endoscopic myotomy.Video 1

There were no adverse events, and the patient was discharged on postoperative day 4, which is standard for patients undergoing POEM at our institution. His symptoms resolved after this treatment. An EGD and barium esophagram were performed 3 months and 1 year after treatment. The EGD showed no food residue, and the barium esophagram demonstrated improved contrast flow.

Endoscopic treatment of Zenkerʼs diverticulum via POEM (Z-POEM) has been reported, but this is the first reported use of the strategy on a post-POEM diverticulum 2 . Given the nomenclature of other endoscopic myotomy procedures, we suggest using “B-POEM” for this therapy (POEM for BOM). We propose B-POEM as a novel treatment for symptomatic BOM.

Endoscopy_UCTN_Code_TTT_1AO_2AN

## References

[1] Triggs JR Krause AJ Carlson DA Blown-out myotomy: an adverse event of laparoscopic Heller myotomy and peroral endoscopic myotomy for achalasia Gastrointest Endosc 2021 93 861 8680 32721488 10.1016/j.gie.2020.07.041 PMC7855725

[2] Elkholy S El-Sherbiny M Delano-Alonso R Peroral endoscopic myotomy as treatment for Zenker's diverticulum (Z-POEM): a multi-center international study Esophagus 2021 18 693 699 10.1007/s10388-020-00809-7 33387150

